# Analysis of lncRNA-miRNA-mRNA expression pattern in heart tissue after total body radiation in a mouse model

**DOI:** 10.1186/s12967-021-02998-w

**Published:** 2021-08-07

**Authors:** Molykutty J. Aryankalayil, Shannon Martello, Michelle A. Bylicky, Sunita Chopra, Jared M. May, Aman Shankardass, Laurel MacMillan, Landy Sun, Jaleal Sanjak, Claire Vanpouille-Box, Iris Eke, C. Norman Coleman

**Affiliations:** 1 Radiation Oncology Branch, Center for Cancer Research, National Cancer Institute, National Institutes of Health, 10 Center Drive, Room B3B406, Bethesda, MD 20892 USA; 2 Gryphon Scientific, Takoma Park, MD 20912 USA; 3 Department of Radiation Oncology, Weill Cornell Medicine, New York, NY 10065 USA; 4 Department of Radiation Oncology, Stanford University School of Medicine, Stanford, CA 94305 USA; 5 Radiation Research Program, National Cancer Institute, National Institutes of Health, Rockville, MD 20850 USA

**Keywords:** Radiation, Biomarkers, Normal tissue injury, miRNA, mRNA, lncRNA, Heart

## Abstract

**Background:**

Radiation therapy is integral to effective thoracic cancer treatments, but its application is limited by sensitivity of critical organs such as the heart. The impacts of acute radiation-induced damage and its chronic effects on normal heart cells are highly relevant in radiotherapy with increasing lifespans of patients. Biomarkers for normal tissue damage after radiation exposure, whether accidental or therapeutic, are being studied as indicators of both acute and delayed effects. Recent research has highlighted the potential importance of RNAs, including messenger RNAs (mRNAs), microRNAs (miRNAs), and long non-coding RNAs (lncRNAs) as biomarkers to assess radiation damage. Understanding changes in mRNA and non-coding RNA expression will elucidate biological pathway changes after radiation.

**Methods:**

To identify significant expression changes in mRNAs, lncRNAs, and miRNAs, we performed whole transcriptome microarray analysis of mouse heart tissue at 48 h after whole-body irradiation with 1, 2, 4, 8, and 12 Gray (Gy). We also validated changes in specific lncRNAs through RT-qPCR. Ingenuity Pathway Analysis (IPA) was used to identify pathways associated with gene expression changes.

**Results:**

We observed sustained increases in lncRNAs and mRNAs, across all doses of radiation. *Alas2*, *Aplnr,* and *Cxc3r1* were the most significantly downregulated mRNAs across all doses. Among the significantly upregulated mRNAs were cell-cycle arrest biomarkers *Gdf15, Cdkn1a,* and *Ckap2.* Additionally, IPA identified significant changes in gene expression relevant to senescence, apoptosis, hemoglobin synthesis, inflammation, and metabolism. LncRNAs *Abhd11os, Pvt1, Trp53cor1*, and *Dino* showed increased expression with increasing doses of radiation. We did not observe any miRNAs with sustained up- or downregulation across all doses, but miR-149-3p, miR-6538, miR-8101, miR-7118-5p, miR-211-3p, and miR-3960 were significantly upregulated after 12 Gy.

**Conclusions:**

Radiation-induced RNA expression changes may be predictive of normal tissue toxicities and may indicate targetable pathways for radiation countermeasure development and improved radiotherapy treatment plans.

**Supplementary Information:**

The online version contains supplementary material available at 10.1186/s12967-021-02998-w.

## Introduction

Radiotherapy (RT) is a mainstay of cancer treatment as it reduces recurrence, improves survival, and enhances the efficacy of other treatments. Ionizing radiation (IR) exposure, whether from RT, diagnostic imaging, or accidental sources (e.g., a nuclear disaster), can cause a multitude of side effects, including secondary cancers and other iatrogenic diseases [1–3]. Accidental or radiotherapeutic normal tissue injury can cause many transient or permanent alterations in both cellular and extracellular components within the irradiated field [4]. These are particularly harmful to critical organs such as the heart. Retrospective studies of atomic bomb survivors found evidence that excess relative risk of death due to heart disease increased by 14% per Gray (Gy) of radiation absorbed, linking radiation exposure with long-term cardiac effects [5–7]. Similarly, liquidators exposed to radiation in the Chernobyl exclusion zone displayed a statistically significant excess relative risk for developing cardiovascular disease [8]. Discovery of organ-specific biomarkers will allow for early treatment prior to clinical manifestations of radiation damage.

In an analysis of breast cancer patients treated with RT, radiation was an independent risk factor for death from cardiovascular disease ten or more years after thoracic radiation [9]. Radiation-induced heart disease (RIHD) is a well-documented side effect of thoracic irradiation during treatment of breast, lung, lymphoma, and other mediastinal tumors [10–13]. Late effects of radiation-induced damage to the heart will become increasingly apparent as the population of long-term cancer survivors continues to increase. By 2022, the U.S. alone will have an estimated 18 million cancer survivors; many of them will have been treated with RT [14, 15].

The first clinical symptom of RT-induced damage to the heart manifests as acute pericarditis between 3 and 6 months after irradiation [16]. However, radiation-induced dysfunction of the heart, including coronary artery disease (CAD), myocardial fibrosis, cardiomyopathy, valvular disease, and arrhythmias leading to congestive heart failure may take decades to manifest [17, 18]. Understanding the molecular mechanism behind RIHD development will help identify efficient prophylactic and mitigative treatments. Furthermore, early detection and prediction of normal tissue injury and cardiotoxicity will facilitate interventions to improve quality of life for RT patients and substantially reduce medical costs related to treatment of secondary diseases.

Radiation-induced DNA damage causes genome-wide transcriptional changes. These changes produce alterations in a wide range of cellular functions from immune response to metabolism [19, 20]. However, prior attempts to discover markers of radiation injury to the heart have been unsuccessful. In a study of patients undergoing thoracic radiation without chemotherapy, analysis of c-reactive protein, angiogenic, and inflammatory markers in serum indicated no correlation between levels and dose of radiation [19]. Other studies on markers of RIHD have yielded conflicting results, with no clear consensus on the value of troponin or brain natriuretic peptides (BNP) levels [21]. One recent study indicated that peroxisome proliferator activator receptor alpha (*Ppara*) may be a dose dependent marker for mitochondrial dysfunction and subsequent RIHD [22]. However, further research is necessary to determine the utility of this marker.

The stability and organ specificity of non-coding RNAs make them attractive as diagnostic and therapeutic biomarkers [23, 24]. Several human and mouse heart RNA expression studies have revealed deregulation of lncRNAs in response to heart damage and disease, with over 600 lncRNAs reported as differentially expressed in clinically failing hearts [25–32]. Previous research has highlighted the importance of miRNAs in diseases for multiple cell types, including cardiomyocytes, endothelial cells, smooth muscle cells, and fibroblasts [33–37]. However, there is limited research on understanding their role in normal tissue damage after radiation [38–40]. Our lab and others have identified alterations in lncRNA and miRNA at long and short time points post-radiation both in vivo and in vitro [41–44]. In a previous study, our laboratory demonstrated dose responsive upregulation in whole blood of damage induced noncoding lncRNA (*Dino*), plasmacytoma variant translocation 1 (*Pvt1*) and tumor protein P53 pathway corepressor 1 (*Trp53cor1*) in a whole-body irradiation mouse model [44]. The results of these studies informed the approach we used in the current investigation.

With cancer and cardiovascular disease as the two leading causes of mortality in the world, understanding the effects of RIHD from RT or accidental exposure will be critical to minimizing health consequences [45]. In this study, we utilized whole transcriptome analysis on mouse heart tissues 48 h after whole-body doses of 1, 2, 4, 8, or 12 Gy. Understanding biological pathways that lead to RIHD development will allow for the identification of treatments to improve quality of life for individuals exposed to radiation, either therapeutically or accidentally, and provide diagnostic and/or prognostic markers of damage.

## Methods

### Total body irradiation of mice and sample collection

Six- to 8-week old female C57BL/6 J mice were given total-body irradiation (TBI) with X-rays using the Small Animal Radiation Research Platform (SARRP Xstrahl Ltd.). Mice were placed in plastic containers and exposed to a single surface dose of 1, 2, 4, 8, or 12 Gy at a dose rate of 1.05 Gy/min. Control mice (0 Gy) were placed in the same type of plastic container and sham irradiated. Three animals per dose were included in the study. Hearts of irradiated and control animals were harvested 48 h after TBI. Organs were snap frozen in liquid nitrogen and stored at − 80 °C until processed for RNA isolation. All animal experiments were performed at the Department of Pathology at New York University (NYU) Langone Medical Center under an approved IACUC protocol as part of a collaborative study.

### RNA isolation

Samples were bathed in liquid nitrogen and pulverized into a fine powder using a mortar and pestle. Approximately 100 µg of powdered sample was lysed with 700 µl of QIAzol lysis buffer (Cat # 79306, QIAGEN) and homogenized by passing the solution through QIAshredder spin columns (Cat # 79654, QIAGEN). RNA isolation was performed using standard miRNeasy mini kit (Cat # 217004, QIAGEN) according to the manufacturer’s protocol. Quality and quantity of the RNA samples were assessed using a DeNovix DS-11 nanodrop spectrophotometer (DeNovix, DE, US) and Agilent Bioanalyzer with the RNA6000 Nano Lab Chip (Agilent Technologies, Santa Clara, CA).

### Microarray analysis

Microarray analysis was performed for sham animals (0 Gy) and 1 Gy, 2 Gy, 4 Gy, 8 Gy, and 12 Gy irradiated animals. Quality assessments and microarray experiments were completed as previously reported [46]. Samples were hybridized to Agilent Mouse GE 8 × 60 K v2 arrays for mRNA expression analysis and to Agilent Mouse miRNA 8 × 60 K v21.0 arrays (Design ID 070155) for miRNA expression analysis. Slides were washed and scanned on an Agilent SureScan Microarray Scanner. Expression values were extracted using Agilent Feature Extraction software and data were analyzed with GeneSpring GX software (Agilent Technologies).

### Real time RT-qPCR analysis of mRNAs and lncRNAs

1000 ng of total RNA was reverse transcribed using RT2 First Strand Synthesis kit (Qiagen, US). Individual RT-qPCR reactions using RT2 qPCR primer assays and RT2 SYBR Green qPCR Master Mix (QIAGEN, US) were performed for the following lncRNAs: *Trp53cor1* (Assay ID No. LPM12776A), *Dino* [47] (FP- GCAATGGTGTGCCTGACTAT; RP- TTCTGGCTTCCCAGAG), Pvt1 (LPM16140A) and *Rplp0* (assay ID no. PPM03561B) in the 48 h mouse heart tissue samples. Relative expression was calculated as: 2^−dCt^ where dCt = Ct [test gene] − Ct [Rplp0] [44]. Following qPCR Primer Assay were used for mRNA validation PPM03371A- Ckap-2, PPM05273A-Alas2, PPM04436C-Gdf15, PPM02901B-Cdkn1a, PPM03154A-Cx3cr1, and PPM04813A-Aplnr. *Rplp0* was used as the normalizing control in both lncRNA and mRNA PCR assays.

### miRNA RT-qPCR

200 ng of total RNA was used for first-strand cDNA synthesis reactions using miRCURY LNA RT Kit (Cat. No. 339340) according to the manufacturers protocol. Reverse-transcription reaction was done at 42° for 60 min, followed by an inactivation step at 95° for 5 min. Quantitative Real-Time PCR was done using individual miRCURY LNA miRNA PCR Assays (Cat. No. 339306) for the following primers (mmu-miR-103a-3p, mmu-miR-149 3p, mmu-miR-211-3p, mmu-miR-3960, mmu-miR-6538, mmu-miR-7118-5p, mmu-miR-8101) to detect differential expression in irradiated vs. control samples. Real time PCR reactions were performed using Applied Biosystems Quant Studio Real-Time PCR machine. PCR steps included initial heat activation at 95° for 2 min followed by two step cycling: Denaturation at 95° for 10 s followed by combined annealing/extension at 56° for 60 s for 40 cycles. A melt curve analysis was performed to ensure the specificity of the corresponding RT-qPCR reactions. Fold change = 2^−ddCt^ where ddCt = dCt (irradiated) − dCt (control); dCt = Ct (gene) − Ct (endo control: UniSp6); and Ct is the threshold cycle number. All assays were performed in triplicates. Statistical significance was calculated using student’s unpaired *t*-test.

### Statistical analysis

Analysis of mRNA and miRNA data was performed using R statistical software and the Bioconductor Linear Model for Microarray Analysis (LIMMA) package in R [48]. Background correction and normalization were performed in R using the normal-exponential correction method and quantile normalization between arrays [49]. Only probes with intensities above background on at least one array were kept in the dataset for analysis. Transcripts with multiple probes were averaged such that the final set reflected best estimates of transcript level expression. A linear model was fit to each probe to assess differential expression for pair-wise dose comparisons within the heart-tissue samples. This method employed an empirical Bayes smoothing approach that results in more stable model estimates by using information on variance from the whole probe set, despite the small number of arrays. Models were developed for each of the pair-wise comparisons between each dose (1, 2, 4, 8, and 12 Gy) and the control probes (0 Gy), and resulting probes were filtered using log_2_ fold change and adjusted p-value thresholds (|log_2_FC| > 1, adjusted p-value < 0.05) [50]. Additionally, a nested interaction model was fit for each probe to examine dose within tissue as a linear (continuous) trend. Each model yielded main effects for the heart tissue and dose within the heart tissue. Probes were filtered using the nested dose coefficients with log fold change and adjusted p-value thresholds (|log_2_FC| > 1, adjusted p-value < 0.05). Finally, gene ontology analysis was utilized to identify affected pathways from the differentially expressed probes.

To identify potential interactions, paired analysis was conducted to evaluate correlative relationships between pairs of differentially expressed mRNA and miRNA probes. mRNA and miRNA probes were paired using shared target transcript Ensembl IDs [51]. Probes that could not be mapped or paired were excluded. Transcripts for miRNA probes were identified using an Agilent microarray gene dataset and the TargetScan database; transcripts for mRNA probes were identified using an Agilent microarray gene dataset [52]. Transcript-miRNA pairs with a TargetScan context++ score above − 1 were excluded. Probe pairs with differentially expressed miRNA and mRNA probes were identified within the heart tissue for continuous dose contrast models. Pearson correlation coefficients of miRNA and mRNA expression across all experiments were calculated and plotted for the differentially expressed probe pairs.

### Ingenuity pathway analysis

Both core and comparison analyses were performed in IPA (QIAGEN Inc., https://www.qiagenbioinformatics.com/products/ingenuitypathway-analysis). Pathways and function terms that satisfied an absolute z-score > 2 and p-value < 0.01 were predicted to be altered based on the gene expression data.

## Results

### Radiation induces widespread transcriptional changes

**Fig. 1:**
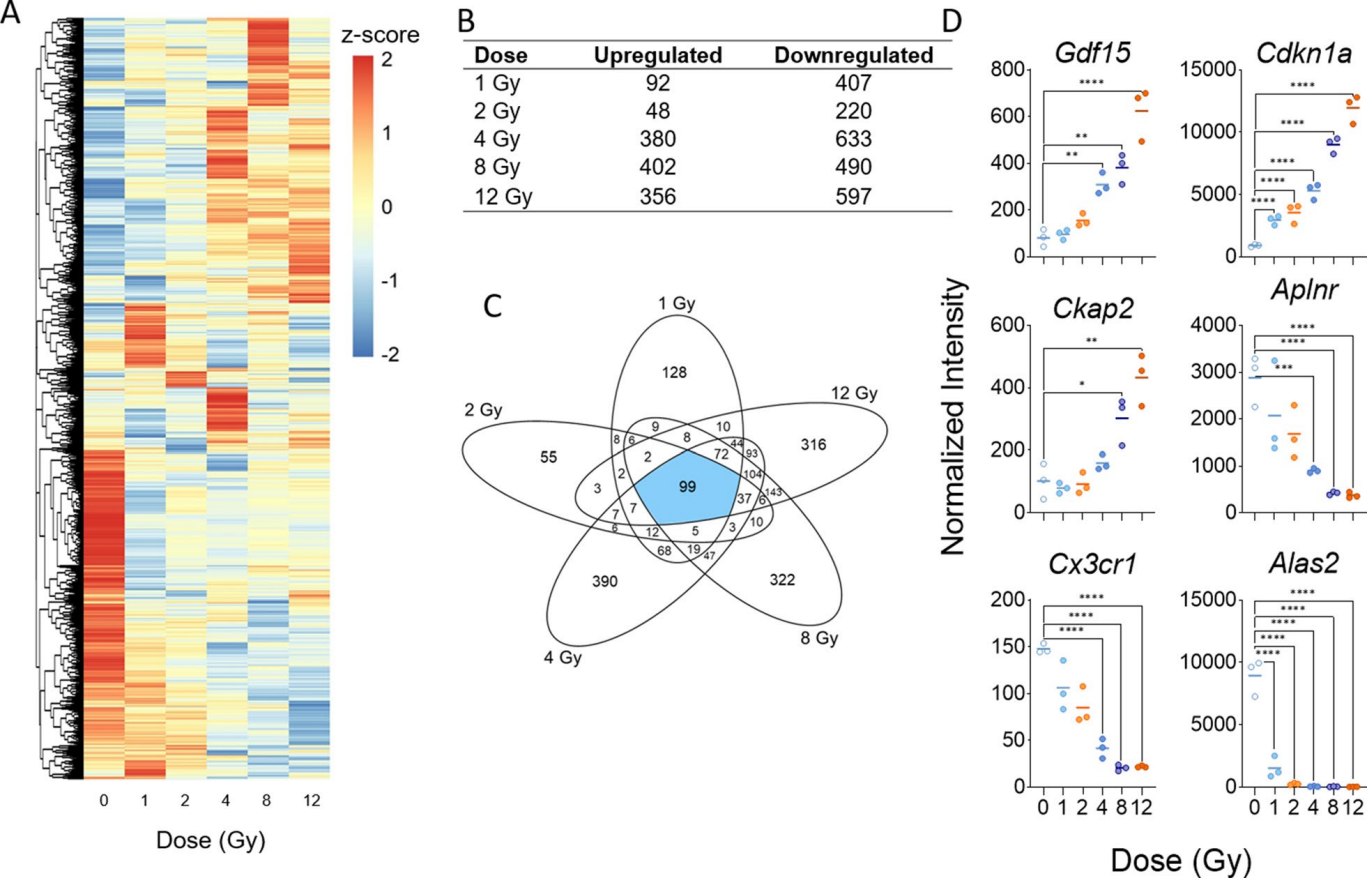
Radiation-induced gene expression profiles in mouse heart tissue. Whole genome microarray analysis was performed on all samples. A linear model was fit to each probe to evaluate differential expression of irradiated samples compared to controls. Criteria of |log_2_Fold Change (FC)| > 1 and Benajmini-Hochberg adjusted (B-H) p-value < 0.05 relative to controls were used to determine significance and differential expression. **A** Heatmap displays expression patterns, represented by z-score, of all differentially expressed mRNAs across all doses and controls. **B** Venn diagram shows dose distribution and overlap of differentially expressed mRNAs across all doses. **C** The number of down-regulated versus up-regulated mRNAs at each dose are shown in the table. **D** Examples of significant linearly up- and down-regulated mRNAs are shown to display the dose response to radiation in heart tissue samples

Microarray analysis performed on all mouse heart samples revealed 2041 differentially expressed genes (|log2FC| > 1; p-value < 0.05) that distinguished unirradiated control samples from samples of at least one dose of TBI mice. Overall, most genes had relatively low to no basal expression in control samples and showed increased expression levels after TBI; however, a cluster of genes showed relatively pronounced high expression in control samples that decreased to low expression after irradiation (Fig. 1A). For each dose, more genes were differentially upregulated than downregulated (Fig. 1B). Across all doses, 99 genes were commonly expressed in response to radiation and 128, 55, 390, 322, and 316 genes were expressed exclusively after 1, 2, 4, 8, and 12 Gy of TBI, respectively (Fig. 1C). Additional file 2: Table S1 lists fold changes and p-values of all differentially expressed genes by dose. While there was no systemic dose–response in terms of the number of genes expressed, we did observe more differentially expressed genes in the higher doses (4, 8, 12 Gy) than in the lower doses (1, 2 Gy). When the dose response of each gene was analyzed by fitting a linear model to each probe, 596 probes were found to have significant dose-responsive up- or down-regulation across all doses; here we present the 20 most upregulated and downregulated genes (Additional file 3: Table S2). *Cdkn1a*, *Ckap2*, and *Gdf15* were among the top 20 probes with the strongest upward linear trend, and *Alas2, Aplnr,* and *Cx3cr1* were among the top 20 probes with the strongest downward linear trend (Fig. 1D). All six genes have previously been reported in the context of radiation or DNA damage response and fall into three main biological roles: cell cycle arrest, hemoglobin metabolism, and inflammatory response (Table 1). The prior relevant literature on *Cdkn1a* [53, 54], *Gdf15* [55, 56], *Ckap2* [57, 58], *Alas2* [59, 60], *Aplnr* [61, 62] and *Cx3cr1* [63, 64] are listed in Table 1. We validated the expression of the most significantly upregulated and downregulated mRNAs in our data using RT-qPCR (Additional file 1: Figure S1).

**Table 1:** Biological roles of most significantly dose-responsive mRNAs

Gene symbol	Gene name	Biological process involvement	Previous reports related to radiation
Cdkn1a	Cyclin-dependent kinase inhibitor 1A	DNA damage-induced apoptosis; cell cycle arrest	[53, 54]
Gdf15	Growth differentiation factor 15	Heart-derived hormone; pathogenesis of atherosclerosis	[55, 56]
Ckap2	Cytoskeleton associated protein 2	Cell cycle arrest; essential for proper chromosome segregation	[57, 58]
Alas2	Aminolevulinic acid synthase 2, erthyroid	Hemoglobin metabolism pathway; cardiotoxicity	[59, 60]
Aplnr	Apelin receptor	Coordination of monocyte trafficking in hemeostatic and inflammatory states	[61, 62]
Cx3cr1	Chemokine (C-X3-C motif) receptor 1	Chemokine binding; cell adhesion	[63, 64]

Genes displayed correspond to the top three most significantly dose-responsive up- and down-regulated mRNAs shown in Fig. 1D. A short-list of biological process involvement and previous reports of involvement in the molecular response to radiation are shown

### Low basal expression levels of long non-coding RNAs in heart tissue showed increased expression after TBI

**Fig. 2:**
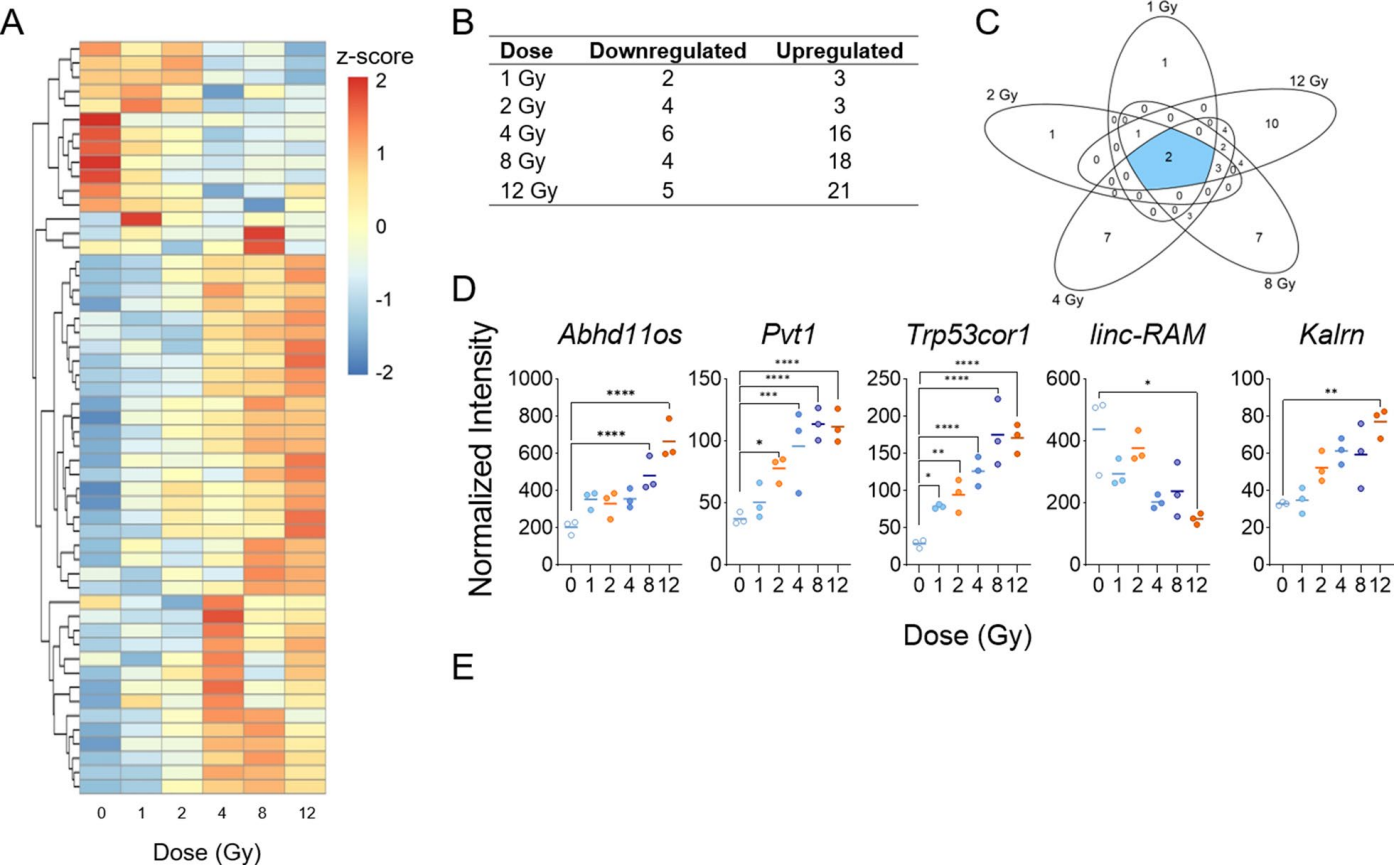
Radiation-induced long non-coding RNA expression profiles in mouse heart tissue. Whole genome microarray data was filtered to include only probes that correspond to transcripts of lncRNAs. A linear model was fit to each lncRNA probe to assess differential expression of irradiated compared to control samples using criteria of |log_2_FC| > 2 and B-H p-value < 0.05. **A** Heatmap displays expression patterns, represented by z-score, of all differentially expressed lncRNAs across all doses and controls. **B** Venn diagram shows dose distribution and overlap of differentially expressed lncRNAs across all doses. **C** The table shows the number of down- versus up-regulated lncRNAs at each dose. **D** Examples of significant linearly up- and down-regulated lncRNAs are shown to display the dose response of lncRNAs to radiation in heart tissue samples. **E** RT-qPCR validation was performed on significantly up-regulated lncRNAs that were previously reported in the blood [44]

To understand the response of heart-based lncRNAs to TBI, we filtered whole genome microarray data to include only probes that correspond to transcripts of lncRNAs. Of the 87 lncRNA transcripts in the microarray data that passed the background intensity cutoff in at least one condition, 46 were differentially expressed in response to radiation, irrespective of the TBI dose (Fig. 2A). Most lncRNAs showed relatively low expression in unirradiated control samples with increased expression after radiation. More probes showed upregulation than downregulation in all doses except 2 Gy, which had 4 downregulated lncRNAs and 3 upregulated lncRNAs (Fig. 2A, B). Two lncRNAs were significantly altered at all doses after radiation, including: chr10:69819062-69871640_F and chr17:29183003-29217681_R (*Trp53cor1-up*) (Additional file 4: Table S3) (Fig. 2C). Additional lncRNAs were altered only at specific doses, with 1, 1, 7, 7, and 10 lncRNAs expressed exclusively in 1, 2, 4, 8, and 12 Gy, respectively (Fig. 2C). Additional file 4: Table S3 lists the fold changes and p-values for the differentially expressed lncRNAs at each dose. Twenty probes showed significant linear upward or downward trends as the dose of TBI increased, demonstrating a linear dose response (Additional file 5: Table S4). *Abhd11os*, *Trp53cor1*, *Pvt1,* and *Kalrn* were among the most significant annotated lncRNAs that became upregulated as radiation dose increased, while the *linc-RAM* (*Malrn*) transcript had the most significant dose-responsive downregulation (Fig. 2D). *Trp53cor1* was the most sensitive to radiation, showing significant increase in the relative intensity in comparison to the unirradiated control even after 1 Gy of TBI. The basal level expression of *Trp53cor1* lncRNA expression was below detection threshold levels in unirradiated heart tissue. Due to this reason, we used relative intensity to describe expression of *Trp53cor1* after radiation.

In contrast, basal expression of *Abhd11os* was at a much higher threshold across all doses, including control samples, with significantly higher expression levels after 8 and 12 Gy TBI. *Pvt1* showed significance after 2 Gy and *Abhd11os* showed significance after 8 Gy, while *Kalrn* and *linc-RAM* showed significance only after 12 Gy TBI. We confirmed the expression of *Dino*, *Pvt1*, and *Trp53cor1* in heart samples through RT-qPCR (Fig. 2E). In concordance with the microarray data, *Trp53cor1* showed very low expression in control samples but significant dose-responsive upregulation after radiation. *Pvt1* also showed consistent results with the microarray in terms of the dose response; however, it showed significance in expression change only after 12 Gy TBI. Damage induced noncoding lncRNA (*Dino*) was not present in our microarray data due to lack of a probe, but prior data from our lab led us to validate its expression in the heart via RT-qPCR. We found that *Dino* is also significantly expressed in the heart after every dose of TBI. Like *Trp53cor1, Dino* showed very low expression levels in control samples but increased significantly after radiation (Fig. 2E).

### Low dose of total-body irradiation induces the most significant and pronounced changes of microRNA expression in mouse heart tissue

**Fig. 3:**
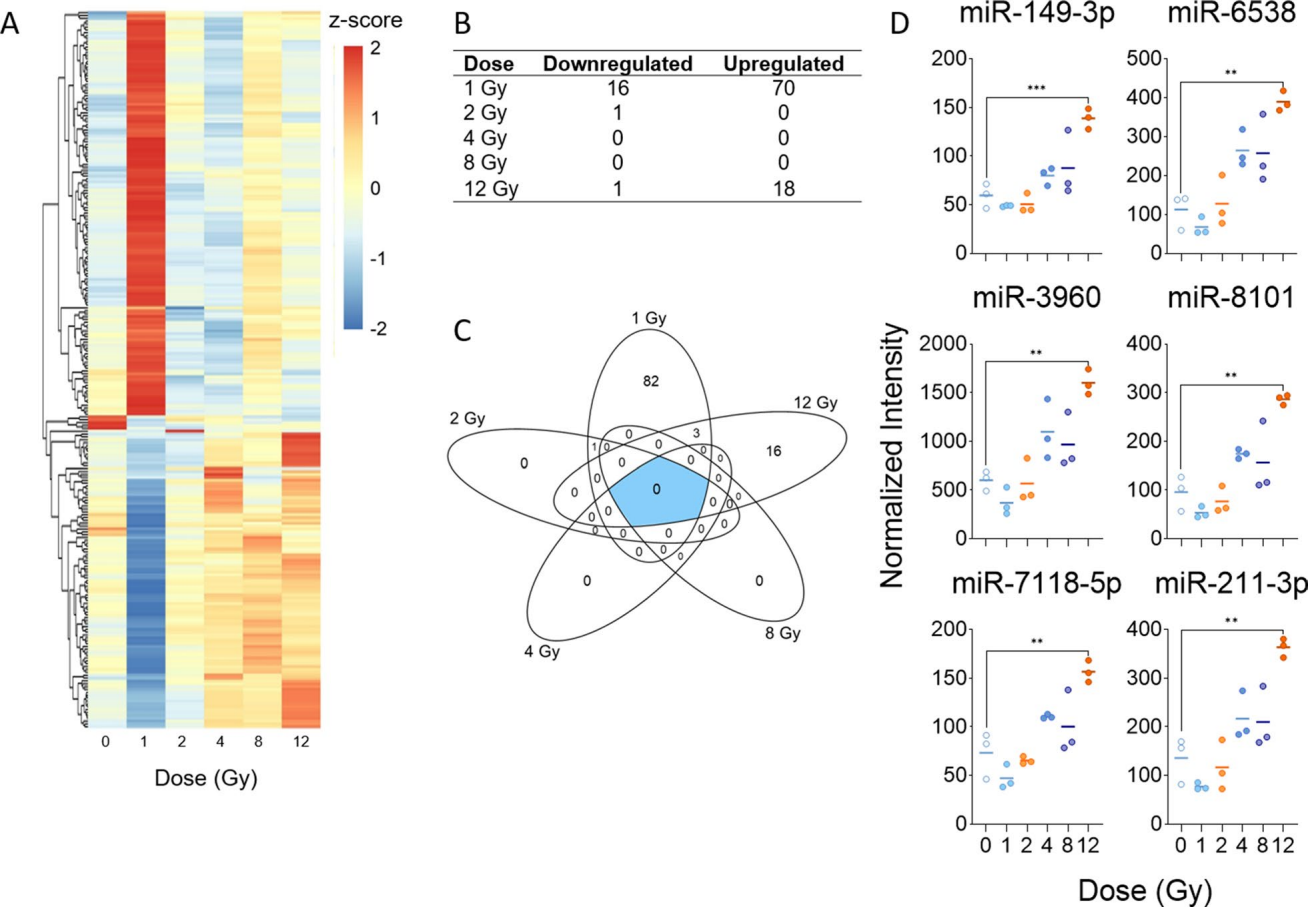
Radiation-induced microRNA expression profiles in mouse heart tissue. Microarray analysis was performed for all samples, and a linear model was fit to each miRNA probe to assess differential expression of irradiated samples compared to controls. Criteria of |log_2_FC| > 2 and B-H p-value < 0.05 relative to controls were used to determine significance and differential expression. **A** Heatmap displays expression patterns, represented by z-score, of all differentially expressed miRNAs across all doses and controls. **B** Venn diagram shows dose distribution and overlap of differentially expressed miRNAs across all doses. **C** The number of down-regulated versus up-regulated miRNAs at each dose are shown in the table. **D** Examples of significant linearly up- and down-regulated miRNAs are shown to display the dose response to radiation in heart tissue samples

A separate whole genome microarray analysis of miRNA expression revealed 102 significantly altered miRNAs in mouse heart tissue in response to radiation. Surprisingly, the largest and most significant changes in expression occurred after 1 Gy of TBI, with 86 differentially expressed miRNAs identified at this dose (Fig. 3A). Furthermore, there were no commonly expressed miRNAs across all doses and no miRNAs significantly expressed in 4 or 8 Gy TBI samples (Fig. 3B, C). We did observe significant regulation of miRNAs at 2 and 12 Gy; however, the numbers were relatively low, with 1 and 19 miRNAs in 2 and 12 Gy, respectively. Fold changes and p-values for all significantly altered miRNAs at each dose are listed in Additional file 6: Table S5. Linear trends were fit to all miRNA probes to identify dose response across all doses and found 21 probes that showed significant upward linear trend (Additional file 7: Table S6). No miRNAs showed a significant downward linear trend. Among the top linear probes were miR-149-3p, miR-6538, miR-3960, miR-8101, miR-7118-5p, and miR-211-3p, all of which showed a statistically significant increase in expression only after 12 Gy of TBI (Fig. 3D). We validated expression of miR-149-3p, miR-3960, miR-7118, miR-6538, miR-8101, and miR-103a-3p (Additional file 8: Figure S2). miR-211-3p was below detection using RT-qPCR (data not shown).

### Pathway analysis of mRNA targets reveals biological role of inversely correlated mRNA-miRNA pairs and their potential as part of an integrated TBI response signature

**Fig. 4:**
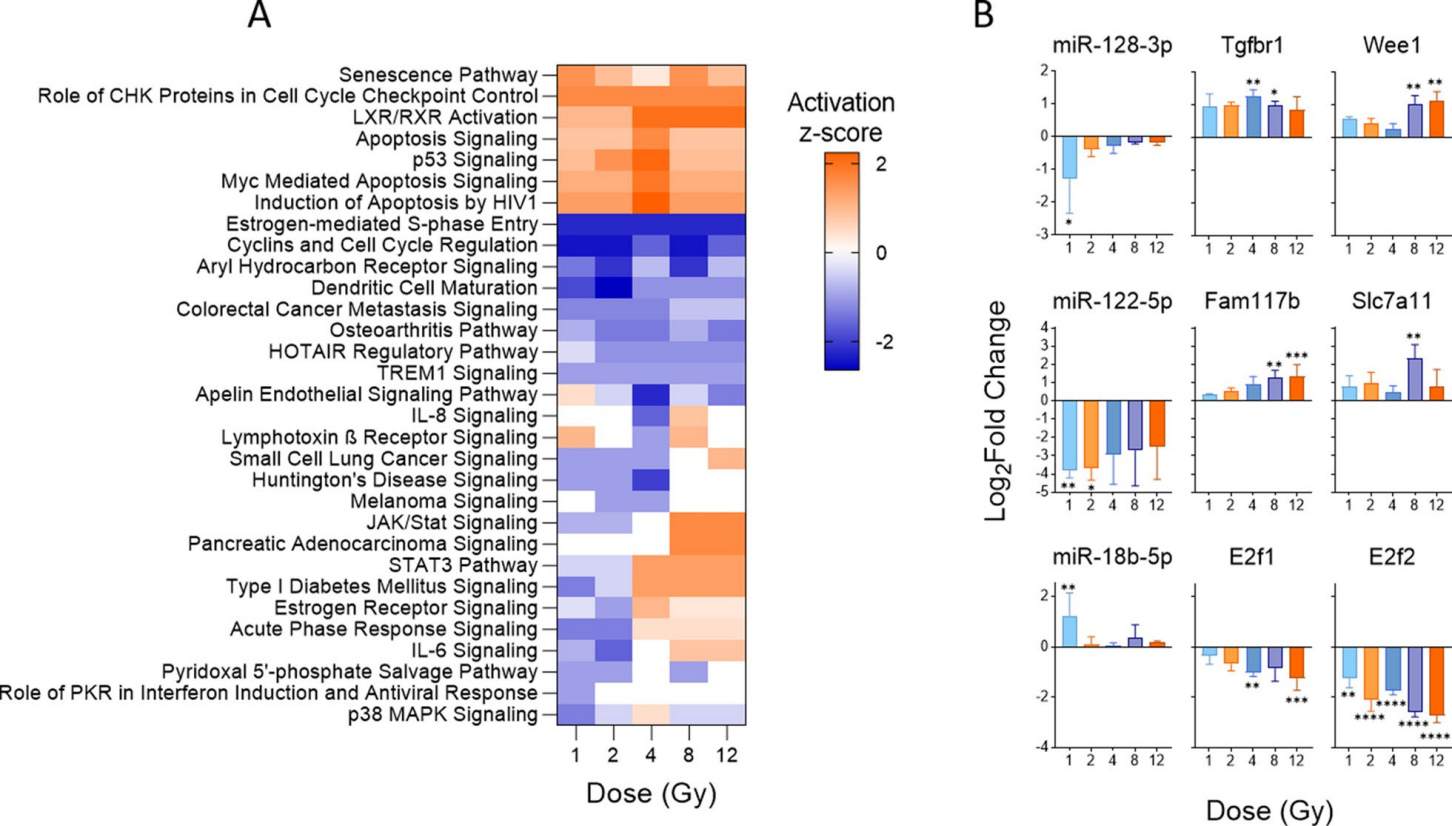
Significant dysregulation of canonical pathways observed through changes in expression of the mRNA targets of differentially expressed miRNAs. Experimentally verified and differentially expressed mRNA targets of differentially expressed miRNAs were analyzed using IPA to predict effects of miRNA-mRNA pairs on canonical pathways. **A** Heatmap displays canonical pathways that were predicted to be significantly dysregulated (B-H p-value < 0.01 across all doses) based upon differentially expressed mRNA targets. A positive z-score indicates predicted activation of the pathway based on gene expression and a negative z-score indicates predicted deactivation of the pathway based on gene expression. Pathways are hierarchically clustered by z-score. **B** Fold changes of inversely correlated miRNA-mRNA target pairs with involvement in the significantly dysregulated pathways. Three miRNAs had two mRNA targets each that were inversely correlated across all doses and significant in at least one condition

Since we are ultimately interested in developing integrated signatures of coding and non-coding RNA response to radiation, we sought to identify potential interactions of the miRNA and mRNA signatures and their biological significance. Using IPA, we first conducted miRNA target filter analysis of the significant differentially expressed miRNAs to identify experimentally verified mRNA targets in our dataset. Canonical pathway analysis of the identified targets revealed significant activation of pathways relevant to cell cycle checkpoint activation and senescence, including p53 signaling and numerous apoptosis signaling pathways, among others (Fig. 4A). Interestingly, most pathways activated across all doses were predicted to have the highest activation after 4 Gy TBI, followed by lower activation in 8 and 12 Gy. One exception to this was the senescence pathway, which showed the lowest activation at 4 Gy. A concurrent higher activation of various apoptosis pathways after 4 Gy TBI may suggest that miRNA-mRNA pairs at this dose exhibit a different response to stress and DNA damage than both lower and higher doses at this time-point after irradiation. Interestingly, analysis of the mRNA targets of the significant miRNAs revealed the strongest activation and deactivation of pathways after 4 Gy TBI, despite a majority of differential miRNA expression after 1 Gy TBI. Since pathway analyses are based on mRNA rather than miRNA data, it is likely that this observation arises from the more pronounced mRNA expression at 4 Gy compared to 1 Gy, After pathway analysis, we identified predicted miRNA-mRNA pairs with inverse expression patterns, demonstrating their potential for inclusion in an integrated RNA marker signature to improve clinical decision making. We found three miRNAs that showed significant expression in at least one dose and had predicted targets with inverse expression patterns, each miRNA with two targets. Radiation decreased expression of both miR-128-3p and miR-122-5p relative to control, while their targets—*Tgfbr1* and *Wee1,* and *Fam117b* and *Slc7a11,* respectively—showed a statistically significant increase in expression as radiation increased (Fig. 4B). The third miRNA, miR-18-5p, showed increased expression after radiation, most significantly after 1 Gy. Its targets, *E2f1* and *E2f2*, showed significant down regulation across all doses.

### Pathway analysis of all mRNAs predicts activation of immune and cell-cycle related pathways and deactivation of metabolic pathways after TBI

**Fig. 5:**
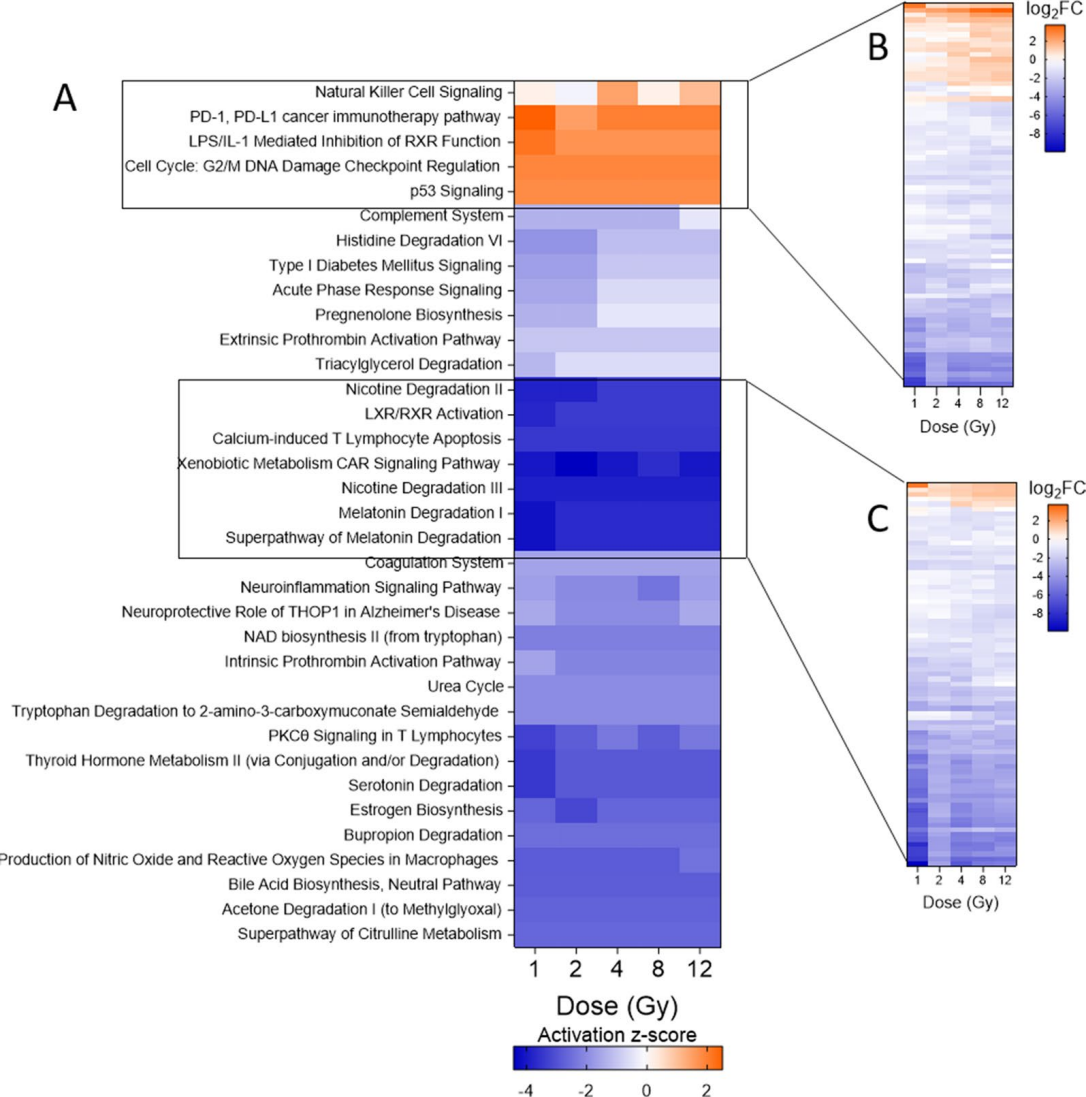
Predicted canonical pathway dysregulation in mouse heart samples based on all differentially expressed mRNAs. IPA was used to perform pathway analysis on all differentially expressed mRNAs to predict pathway involvement, independent of the target relationship with differentially expressed miRNAs. **A** Heatmap displays the top 35 most significantly dysregulated pathways (B-H p-value < 0.01). A positive z-score indicates predicted activation of the pathway based on gene expression and a negative z-score indicates predicted deactivation of the pathway based on gene expression. Pathways are hierarchically clustered by z-score. **B** Heatmap shows the log_2_FC of the 78 differentially expressed genes with involvement in the cluster of activated pathways. **C** Heatmap shows the log_2_FC of 79 differentially expressed genes with involvement in the cluster of most deactivated pathways

While understanding the interactions and biological implications of the miRNAs and mRNAs is critical for developing an integrated biomarker signature, we hypothesized that because the relatively low number of differentially expressed miRNAs would limit the number of mRNAs included in the pathway analysis, we could potentially miss genes that play a significant role in pathway regulation. Therefore, we also conducted a canonical pathway analysis using all differentially expressed mRNAs, irrespective of interactions with miRNAs in our dataset. A similar overall pattern of pathway regulation exists between the target mRNAs and target/non-target mRNAs, with significant deactivation of most of the pathways involved (Fig. 5A). Several pathways related to coagulation, including both ex- and intrinsic prothrombin activation and the coagulation system pathway, were downregulated across all doses. Changes in immune-related pathways were less consistent in terms of activation or deactivation. While natural killer cell signaling was activated significantly after 4 and 12 Gy TBI, the complement system was inhibited across all doses.

Two clusters of pathways showed the most pronounced activation and deactivation. Five pathways were predicted to be activated across all doses, and all were involved in immune response or cell cycle regulation. There were 78 genes differentially expressed in these pathways, a majority of which were downregulated with respect to the control (Fig. 5B). A full list of genes can be found in Additional file 9: Table S7. In contrast to the pathway analysis of the mRNA targets, which showed the highest activation of several pathways after 4 Gy TBI, all activated pathways except natural killer cell signaling were consistently activated as the dose increased. We observed that radiation inhibits pathways relevant to xenobiotic metabolism and biosynthesis of lipids, including hormones. This is demonstrated by activation of LPS/IL-1 mediated inhibition of RXR function and inhibition of the super-pathway of melatonin degradation, among others. There were 79 genes involved in deactivation of these seven pathways, most of which were downregulated after TBI (Fig. 5C, Additional file 9: Table S7).

### TBI deactivates metabolic pathways and alters the expression of metabolism-related genes in heart tissue

**Fig. 6:**
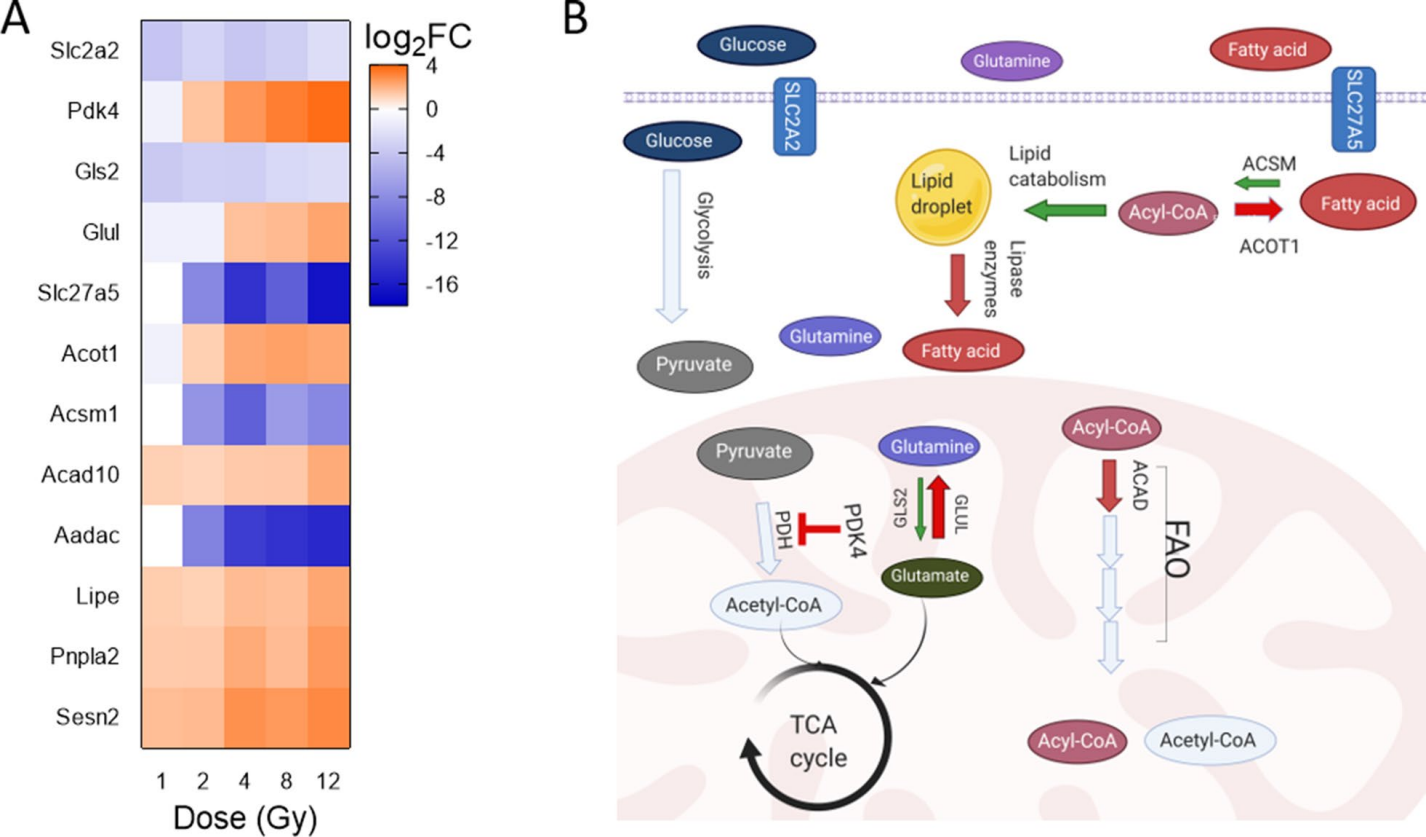
Differentially expressed genes involved in metabolic pathways suggest impact of radiation on metabolism in the heart. Differentially expressed genes with involvement in metabolic signaling pathways were identified using IPA. **A** Heatmap displays the log_2_FC of the genes at each dose. **B** Cartoon depicts changes predicted based upon the genes shown in (**A**). Red arrows indicate increased gene expression. Green arrows indicate decreased expression of gene. Light blue indicates no significant change to gene expression

IPA analysis predicted significant deactivation of several metabolic pathways, such as triacylglycerol degradation and type I diabetes mellitus signaling, among others (Fig. 5A). To further understand how radiation alters metabolism, we used IPA to filter the microarray gene expression data to include only the significantly differentially expressed genes with involvement in metabolic energy production pathways, with emphasis on fatty acid oxidation (Fig. 6A). Of note, there was a significant downregulation of solute carrier 2a2 (*Slc2a2*) and concurrent upregulation of pyruvate dehydrogenase kinase 4 (*Pdk4*), especially after 4 Gy TBI. Similarly, as dose increased, we observed upregulation of glutamine synthetase (*Glul*), which encourages conversion of glutamate to glutamine. We also observed downregulation of glutaminase (*Gls2*), which converts glutamine to glutamate.

Gene expression data for fatty acid metabolism at 48 h post radiation was contradictory. We observed a downregulation of solute carrier 27a5 (*Slc27a5*), which is associated with fatty acid entry into the cell; an upregulation of sestrin 2, which inhibits lipid catabolism; and an upregulation of hormone sensitive lipase (*Lipe*) and adipose triglyceride lipase (*Atgl* or *Pnpla2*), which cleave lipid droplets to allow use of fatty acids in FAO. However, there was a downregulation of arylacetamide deacetylase (*Aadac*) which shares homology with *Lipe* and is also thought to control triglyceride levels. Additionally, acyl-CoA synthetase medium chain family members 1 (*Acsm1*) was downregulated, while acyl-CoA thioesterases 1 (*Acot1*) and acyl-coA dehydrogenase 10 (*Acad10*) were notably upregulated as radiation doses increased.

## Discussion

Normal tissue damage of the heart is a clinically relevant problem in both therapeutic and accidental exposure to radiation. A previous study highlighted the long term impact of radiation on male macaques, which showed significant myocardial fibrosis and smaller cardiac dimensions at 5.6–9.7 years post radiation exposure with 6.5–8.4 Gy [65]. Historical data for macaques receiving TBI, the LD_50/30_ post X-ray irradiation varies between 4.92 and 7.18 Gy [66]. In contrast, LD_50/30_ is approximately 7.2 Gy in female mice receiving TBI X-ray [67]. Our study focused on short term (48 h) changes in gene expression after TBI doses in mice. Understanding mechanisms and markers of radiation injury at an early time-point following exposure can improve methods to mitigate long-term damage and death. Recent research in the radiation biodosimetry field indicates the importance of looking for alterations in multiple biomarkers rather than relying on a single marker [68, 69]. To this end, we have identified changes in multiple mRNAs, miRNAs, and lncRNAs across doses to provide potential markers of tissue damage.

### TBI dysregulates pathways relevant to cell cycle arrest, hemoglobin synthesis and immune response in the heart

The observed early changes in gene expression led to significant dysregulation of pathways commonly associated with radiation exposure, including cell cycle arrest, apoptosis, and senescence. The most significantly altered genes, *Cdkn1a* and *Ckap2*, are capable of inducing cell cycle arrest or apoptosis under stress conditions [70, 71]. The observed upregulation of *Gdf15* and downregulation of *Aplnr* are associated with stress induced senescence and have previously been reported as biomarkers of radiation exposure [56, 72, 73]. The inversely correlated expression of miRNAs and their mRNA targets that are associated with these pathways provides insight into the potential mechanisms of acute effects of TBI and targets to mitigate acute and delayed effects on heart tissue. Prior research demonstrated miR-128 negatively regulated *Wee1* and *Tgfbr1*, genes involved in mitotic inhibition [74–76]. Additionally, miR-122 is known to increase radiation sensitivity and targets *Slca11* and *FAM117b* [77]. While little is known about the function of *Fam117b*, *Slc7a11* downregulation is associated with RT-induced ferroptosis in tumor cells [78, 79]. The miR-17-92 cluster represses *E2F1-3* to regulate cell proliferation and apoptosis, and includes miR-18a [80]. A negative feedback loop has been observed within this cluster, as *E2F1* upregulates miR-17-92 which causes increased repression of the genes *E2F1-3* [81]. Mechanistic studies are required to confirm the miRNA-mRNA interdependent functions in our data. Similarly, further research into lncRNA-mRNA functions are also needed. Acute lncRNA *Dino* overexpression caused an increase in *Cdkn1a* expression in cervical cancer cells [82]. Our data suggests an interplay between both lncRNA *Dino* and mRNA *Cdkn1a* expression.

Anemia and decreased hemoglobin levels are a known side effect of RT [83]. We observed decreased expression levels of genes relevant to hemoglobin synthesis. *Alas2*, the rate limiting enzyme in heme synthesis, became increasingly downregulated as radiation dose increased. Similar downregulation of *Alas2* was recently cited as a potential predictive marker for radiation induced hematological toxicity in cancer patients [60]. Other genes associated with hemoglobin synthesis, including *Hbb-bt*, *Hbb-b1*, and *Hba-a1,* were also significantly, linearly downregulated (Additional file 3: Table S2).

Our pathway analysis also indicated inflammatory pathways are downregulated at higher dose levels at 48 h post-radiation. Of note, TBI induced significant downregulation of *Cx3cr1*, which is known to induce recruitment of immune cells and an inflammatory response in smooth muscle and endothelial cells [84]. Short-term data contrasts long-term in vivo data that showed upregulation of inflammatory markers in the heart of a mouse model 40 days post-irradiation [17]. Additional studies that include intermediate timepoints are needed to clarify when the heart transitions from an anti-inflammatory to a pro-inflammatory response after radiation to enable improved treatment options.

Consistent with previous research [85], our findings indicated inhibition of extrinsic and intrinsic prothrombic pathways that couple with inhibition of coagulation pathway after radiation exposure. Radiation is known to increase likelihood of coagulopathy, which can lead to death when untreated [86]. We also observed downregulation of hemoglobin subunit beta (*Hbb*) and hemoglobin subunit alpha (*Hba*) (Additional file 3: Table S2). In combination with anemia, failure to clot produces hemostatic dysfunction and potential death, though the pathogenesis is poorly understood [87]. Our research highlights these alterations in gene expression to provide insight into potential mechanisms of and therapeutic targets for acute radiation syndrome (ARS).

Retinoid X receptor and liver X receptor (RXR/LXR) activation is associated with protection against heart failure due to their role in improving glucose tolerance, decreasing lipid accumulation, and decreasing inflammation [88]. RXR signaling has previously been shown to increase estradiol synthesis from pregnenolone [89]. We observed that the estrogen and pregnenolone biosynthesis pathways are also inhibited. Inhibition of RXR/LXR and its downstream pathways coupled with inhibition of triacylglycerol degradation may indicate a deleterious increase in lipid accumulation within the heart.

### Radiation induced lncRNA and miRNA provide potential insight into RIHD through signaling pathways

Previously, we reported the concomitant differential expression of p53-related lncRNAs such as *Pvt1*, *Dino*, *Trp3cor1* after TBI in a mouse model [44]. In the current study, we observed significant alterations in abhydrolase domain containing 11, opposite strand (*Abhd11os*), *Pvt1*, *Trp3cor1*, *Kalrn*, linc-RNA activator of myogenesis (*Linc-RAM*), lncRNA chr10:69819062-69871640_F and *Dino*. Increased expression of *Abhd11os* has previously been shown to decrease lesion size in a Huntington’s disease mouse model and the authors reported its crucial roles in neurodegenerative diseases [90], but the exact mechanism is still unclear. *Linc-RAM* encourages adult skeletal muscle stem cells to differentiate into skeletal muscle through myogenic differentiation (MyoD), which aids in muscle repair after injury [91, 92]. While *Linc-RAM* has not been directly associated with cardiomyocytes, MyoD-null dystrophin-null transgenic mice develop severe cardiomyopathy [93]. *Pvt1* has been associated with radiation resistance in cancer cells and cardiac hypertrophy in cardiomyocytes [94–96]. Previous studies showed that *Pvt1* binds *Cdkn1a* and miR-149-3p to suppress their activity in primary chondrocytes and Burkitt lymphoma Rajit cells, respectively [97, 98]. In contrast, *Gdf15* is a downstream target of *Pvt1* and was positively regulated by the lncRNA in hepatocellular carcinoma cells [99]. Our data indicates an increase in expression of *Pvt1*, miR-149-3p, *Cdkn1a* and *Gdf15* by 12 Gy, suggesting that the interactions between these noncoding and coding RNA must be further elucidated in normal tissue. Further, the clinical relevance of lncRNA chr10:69819062-69871640_F has yet to reported. With the observed significant expression changes across all doses of radiation, further studies are crucial to inform more conclusions about the role of this lncRNA in the heart’s response to radiation.

Prior research demonstrated the integral role that miRNAs play in cardiac fibrosis and proliferation as well as response to radiation injury [100, 101]. We therefore anticipated changes in miRNA expression in heart tissue after TBI. Surprisingly, and possibly due to the early time point and stringent statistical analysis, we only observed significant upregulation of miRNAs at 1 Gy and 12 Gy. Among the up-regulated miRNAs, miR-149-3p has previously been implicated in multiple functions, including cell migration repression and metabolic modifications in A549 cells, a non-small cell lung carcinoma (NSCLC) model [102]. Additionally, miR-211 has been demonstrated to decrease cell proliferation and metastasis in vitro in breast and renal cell carcinoma models [103, 104], while miR-3960 has been implicated in calcification, decreased elasticity, and cardiac dysfunction in vascular smooth muscle cells of male C57BL/6 mouse aortas [105]. Arterial calcification and valvular, ventricular, and diastolic dysfunction are well-known complications of RIHD disease [106]. While the functions of certain miRNAs are not well understood, previous studies indicate that miR-8101 and miR-6538 are associated with heart failure [107, 108].

### TBI causes miRNA and mRNA expression changes that may indicate similar pathogenesis of end-stage heart failure

Upregulation of miR-149-3p is associated with inhibition of glucose metabolism. Its role as a therapeutic target to protect against diet-induced obesity and metabolic dysfunctions was shown previously in both colorectal cancer patients, tumors taken from colorectal cancer patients and male C57BL/6 J [109, 110]*.* In general, the observed changes in metabolism-related gene expression suggest that fatty acids are not being used for catabolism (Fig. 6B). Increasing doses of radiation appear to inhibit glucose oxidation through increased expression of *Pdk4,* which uncouples glycolysis from oxidative phosphorylation by blocking pyruvate dehydrogenase (*Pdh*), and decreased expression of *Acsm1* [111, 112]. However, we also observed a significant downregulation of the transporter *Slc27a5,* which would inhibit entry of fatty acids into the cell for anabolic or catabolic use. This could be a fatal side effect of IR exposure because the adult heart relies on fatty acid oxidation as its main source of energy production [113]. Since *Lipe* and *Pnpla2* were upregulated, the heart may be relying on internal stores of triacylglycerol to produce energy. Aside from *Acsm1*, no overall changes in acyl-CoA synthetases were observed; these enzymes combine fatty acids with Coenzyme A for use in FAO or lipogenesis. We observed upregulation of *Acot1* which plays contradictory roles in FAO as it can separate long chain fatty acids from coenzyme A to decrease the available substrate pool [114]. However, prior research indicates *Acot1* also increases FAO through activation of peroxisome proliferator-activated receptor α (*Ppar-α*), which upregulates *Acad* variants [115–117]. We only observed an upregulation of *Acad10* and there was no significant change to *Ppar-α* or other *Acad* variants at 48 h after radiation. Furthermore, there was no change in expression of other genes within the FAO pathway. This inhibition of glucose oxidation paired with an apparent reliance on FAO and an increase in free fatty acids matches what is seen in some forms of end stage heart failure [118, 119].

### Gene expression changes in TBI C57Bl/6 match those identified in previous study of TBI Gottingen minipigs

A recent study from our lab reported survival predictive signatures inherent to heart, lung and liver in TBI Gottingen minipigs [120]. Increased expression of *Pdk4* was significantly upregulated in the hearts of non-surviving (survived < 7 days post-TBI) minipigs. We also observed increased expression of *Pdk4* at 4–12 Gy radiation. As previously mentioned, the upregulation of *Pdk4* is associated with decreased glucose oxidation and potential failure of energy production. Hearts from these non-surviving minipigs showed a decrease in the Apelin signaling pathway when compared to survivors and sham animals. In the present study, we also observed inhibition of the Apelin signaling pathway starting at 2 Gy. Apelin signaling is important for endothelial cell proliferation and migration and has been shown to inhibit TGF-β induced cardiac fibrosis and senescence [121]. Conversely, downregulation of Apelin has been linked to heart failure and ventricular dysfunction [122]. Both these findings implicate centrality of the endothelial cell damage in radiation induced heart injury. Finding consistency between mouse and minipig studies indicates conservation of the radiation response across species, suggesting that rescuing the function of this pathway may prevent RIHD in humans as well.

### Similarities in TBI-induced gene expression between heart and blood samples indicates potential biomarkers for effective triaging with radiation biodosimetry

Finding normal tissue injury markers in a less invasive way is warranted for clinical applications. From this perspective, we looked at the commonality of gene and lncRNA expression changes between heart tissue of the current study and mouse whole blood after TBI from a study previously published by our group [44]. Interestingly, we detected few genes in common between both mouse heart tissue and whole blood. We focused on genes altered at 48 h after 8 Gy in whole blood and compared these alterations to changes found in our current study of mouse heart tissue. Significantly altered genes included *Cdkn1a*, *Pmaip1*, *H2Aa*, *H2Ba1*, *Cx3cr1*, *Snca*, and *Gm9992* (Additional file 10: Table S8). Another previous study from our lab indicated that *Pvt1* was significantly expressed in whole blood as early as 16 h after at least 2 Gy TBI, and was sustained until 48 h after TBI [44]. Similarly, at 48 h post-TBI, *Trp53cor1* and *Dino* were significantly upregulated in the whole blood of 12 Gy and 8 Gy TBI mice, respectively. We observed similar expression patterns of these lncRNAs in heart samples after 48 h.

We also compared the heart data to a previously reported in vitro study of gene expression changes in human coronary artery endothelial cells (HCAEC) at 24 h after 10 Gy of single dose radiation [42]. The genes *Cdkn1a,* growth differentiation factor 15 (*Gdf15*), and DNA damage-inducible transcript 4 (*Ddit4/Redd1)* have previously been reported as radiation markers [72, 123, 124]. They showed concomitant upregulation in mouse heart tissue (Additional file 10: Table S8) and in HCAEC [42]. Additionally, we noted the upregulation of hypoxia-inducible factor 3a (*Hif3a*) and insulin-like growth factor 1 (*Igf1*) in mouse heart tissue, whereas these two genes showed significant downregulation in HCAEC in vitro study. In rat cardiomyocytes, *Hif3a* silencing led to increased cell viability and decreased necrosis after hypoxia challenge, suggesting decreased *Hif3a* expression is cardioprotective [125]. In a population study of elderly individuals, decreased serum *Igf1* expression was shown to be a risk factor for mortality after ischemic heart disease [126]. These regulation inconsistencies may stem from differences between models, time points, and radiation dose rate between in vivo and in vitro experiments.

### Future directions

With the use of thoracic RT and the continued risk of a large-scale nuclear exposure incident inadvertently causing potential damage to the heart, understanding the effects of IR exposure on critical organs such as the heart will improve patient outcomes. For clinical management, early biomarkers could be predictive of later damage, enabling alteration of the dose to the organs at risk, use of medical countermeasures, or implementation of an appropriate long-term medical management strategy. For a nuclear exposure incident, the dose will have been delivered such that the injury falls within delayed effects of acute radiation exposure (DEARE), but the mitigator and medical-management approach would still be relevant.

In addition to identifying blood-based signatures for rapid triaging, we are also working on identifying expression changes within organs (e.g., heart, lungs, liver) affected by radiation to predict both short- and long-term organ injury. It is therefore clinically relevant to determine the response of the markers to fractionated radiation and in the presence of pre-existing conditions. Palayoor et al. showed more pronounced miRNA and mRNA expression changes in an in vitro HCAEC model after multifractionated radiation compared to a single dose of radiation suggesting that we may see similar trends in vivo [42]. Additionally, a study of acute lymphocytic leukemia patients that received six fractions of 2 Gy TBI showed a significant and sustained increase in blood-based *Cdkn1a* after each fraction [127]. Blood from these pediatric cancer patients also showed higher baseline levels of *Cdkn1a* expression compared to healthy controls, demonstrating the importance of addressing the effects of confounding factors on expression changes. Recognizing that heart biopsies would not be a suitable method to triage patients in an exposure scenario, we are also currently investigating the short- and long-term circulating RNA response of non-human primates exposed to whole thorax irradiation. This would not only enable monitoring of organ-specific damage sustained during accidental exposures but would also have applications in predicting normal tissue toxicity as a side-effect of RT for the treatment of cancer.

## Supplementary Information

**Additional file 1: Figure S1. RT-qPCR validation of mRNA biomarker expression in heart tissue.** The microarray results were confirmed by RT-qPCR for *Aplnr*, *Gdf15*, *Cdkn1a*, *Ckap2*, *Cx3cr1*, and *Alas2*. Fold change values relative to 0 Gy are shown for the three samples each at 1, 2, 4, 8, and 12 Gy. An asterisk (*) indicates statistically significant value by student t-test, comparing control to irradiated sample (p-value < 0.05).

**Additional file 2: Table S1. Differentially expressed genes at each dose compared to controls.** Each tab corresponds to a specific dose. Columns B through G list the Agilent probe ID, systematic name (ID of target sequence designed to hybridize with the Agilent probe), p-value, Benjamini–Hochberg adjusted p-value, B statistic (log of the odds that the gene is differentially expressed), and the average log_2_FC across samples exposed to the dose, respectively. Probes are listed in order of decreasing significance.

**Additional file 3: Table S2. Top 20 most significantly up- and down-regulated mRNAs that showed a linear trend across all doses.** Tables of up-regulated (A) and down-regulated (B) linear, or dose-responsive, mRNAs. Gene symbol, systematic name, Benjamini–Hochberg adjusted p-value, B statistic, and the average log_2_FC across all doses are shown in each table. Genes are listed in order of decreasing significance.

**Additional file 4: Table S3. Differentially expressed lncRNAs at each dose compared to controls.** Each tab corresponds to a specific dose. Columns B through G list the Agilent probe ID, systematic name, p-value, Benjamini–Hochberg adjusted p-value, B statistic, and the average log_2_FC across samples exposed to the dose, respectively. Probes are listed in order of decreasing significance.

**Additional file 5: Table S4. Significantly up- and down-regulated lncRNAs that showed a linear trend across all doses.** Table of up- and down-regulated linear, or dose-responsive, lncRNAs. lncRNA symbol (if annotated), systematic name, Benjamini–Hochberg adjusted p-value, B statistic, and the average log_2_FC across all doses are shown in each table. For unannotated lncRNAs, a BLAT analysis was performed using ensmbl.org and the overlapping transcript, if any, was listed in the table. The asterisk (*) denotes the transcripts identified through BLAT. lncRNAs are listed in order of decreasing significance.

**Additional file 6: Table S5. Differentially expressed miRNAs at each dose compared to controls.** Each tab corresponds to miRNAs that were significantly differentially expressed at the specific dose. Columns B through G list the Agilent probe ID, systematic name, p-value, Benjamini–Hochberg adjusted p-value, B statistic, and the average log_2_FC across samples exposed to the dose, respectively. Probes are listed in order of decreasing significance.

**Additional file 7: Table S6. Top 20 most significant miRNAs that showed a linear trend across all doses.** Table of up-regulated linear, or dose-responsive, miRNAs. lncRNA symbol (if annotated), systematic name, Benjamini–Hochberg adjusted p-value, B statistic, and the average log_2_FC across all doses are shown in each table. For unannotated lncRNAs, a BLAT analysis was performed using ensmbl.org and the overlapping transcript, if any, was listed in the table. The asterisk (*) denotes the transcripts identified through BLAT. lncRNAs are listed in order of decreasing significance.

**Additional file 8: Figure S2. RT-qPCR validation of miRNA biomarker expression in heart tissue.** The microarray results were confirmed by RT-qPCR for miR-149-3p, miR-103a-3p, miR-6538, miR-3960, miR-7118, and miR-8101. Fold change values relative to 0 Gy are shown for the three samples each at 1, 2, 4, 8, and 12 Gy. An asterisk (*) indicates statistically significant value by student t-test, comparing control to irradiated sample (p-value < 0.05).

**Additional file 9: Table S7. Differentially expressed genes with involvement in the most activated and most deactivated pathways.** A list of differentially expressed genes and the corresponding log_2_FC at each dose are included in each tab. The 78 genes in the activated pathways are listed in the first tab, and the 79 genes in the deactivated pathways are listed in the second tab.

**Additional file 10: Table S8.** Top 20 probes differentially expressed in a dose dependent manner in the heart samples compared to differentially expressed genes from whole blood collected from the same animals at 48 h timepoint and also compared to Human Coronary Artery Endothelial cells exposed to 10 Gy radiation at 24 h timepoints.

## Data Availability

Research data and material are stored in an institutional repository and will be shared upon request to the corresponding author.

## Abbreviations

RT: Radiotherapy
IR: Ionizing radiation
miRNAs: MicroRNAs
lncRNAs: Long non-coding RNAs
mRNAs: Messenger RNAs
