# Supplementary figures and images for: Analysis of lncRNA-miRNA-mRNA expression pattern in heart tissue after total body radiation in a mouse model
Source: J Transl Med. 2021 Aug 7;19:336. doi: 10.1186/s12967-021-02998-w (PMC8349067; doi:10.1186/s12967-021-02998-w)

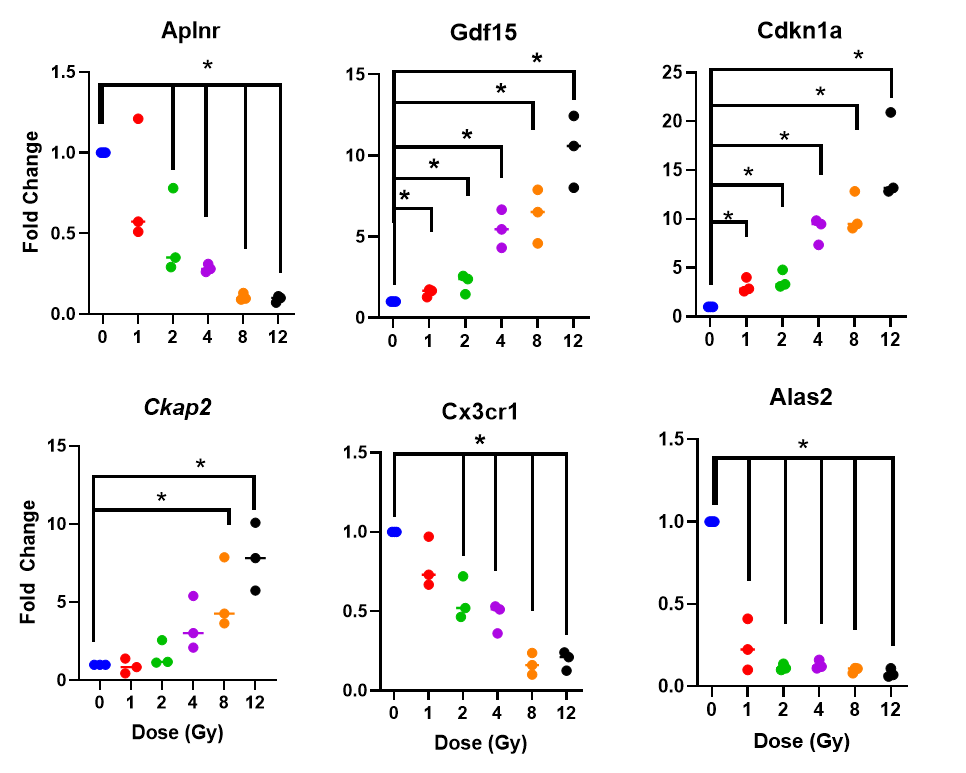

Supplement: Supplementary file 1 — Additional file 1: Figure S1. RT-qPCR validation of mRNA biomarker expression in heart tissue. The microarray results were confirmed by RT-qPCR for Aplnr, Gdf15, Cdkn1a, Ckap2, Cx3cr1, and Alas2. Fold change values relative to 0 Gy are shown for the three samples each at 1, 2, 4, 8, and 12 Gy. An asterisk (*) indicates statistically significant value by student t-test, comparing control to irradiated sample (p-value < 0.05). [file 12967_2021_2998_MOESM1_ESM.png]

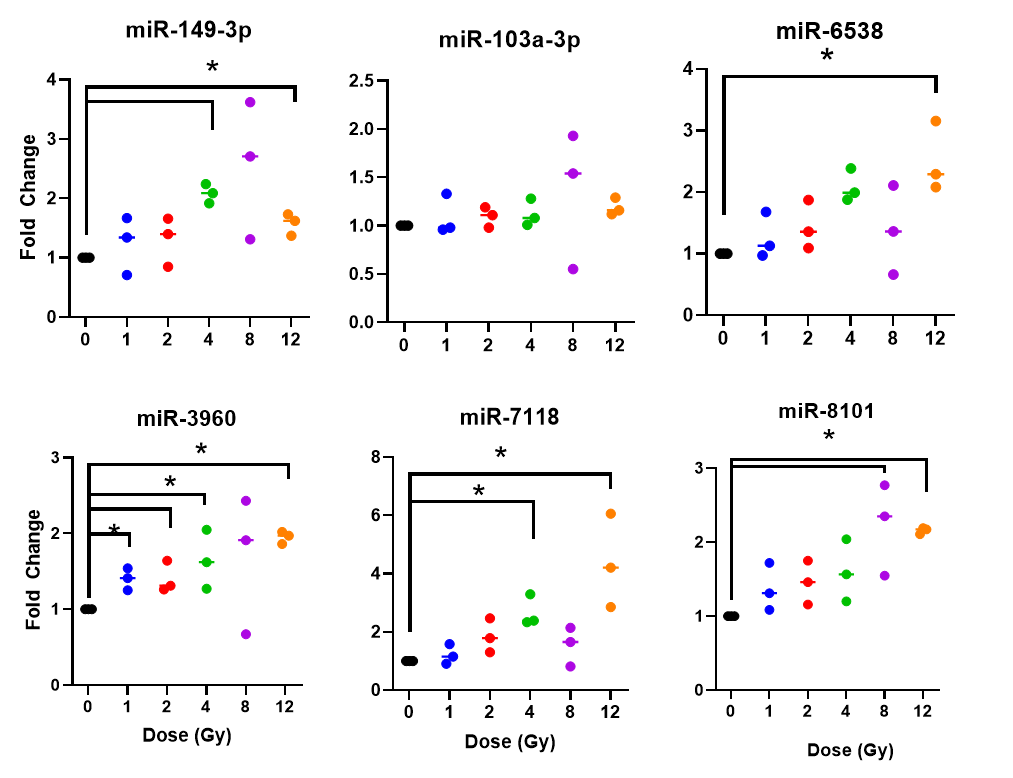

Supplement: Supplementary file 8 — Additional file 8: Figure S2. RT-qPCR validation of miRNA biomarker expression in heart tissue. The microarray results were confirmed by RT-qPCR for miR-149-3p, miR-103a-3p, miR-6538, miR-3960, miR-7118, and miR-8101. Fold change values relative to 0 Gy are shown for the three samples each at 1, 2, 4, 8, and 12 Gy. An asterisk (*) indicates statistically significant value by student t-test, comparing control to irradiated sample (p-value < 0.05). [file 12967_2021_2998_MOESM8_ESM.png]
